# Inspiration to mRNA-based COVID-19 vaccination: Serious adverse case reports with hepatitis B vaccine in real-world

**DOI:** 10.3389/fped.2022.888686

**Published:** 2022-09-23

**Authors:** Jinmiao Lu, Xunjie Zhang, Hong Xu, Zhiping Li

**Affiliations:** ^1^Department of Clinical Pharmacy, National Children's Medical Center, Children's Hospital of Fudan University, Shanghai, China; ^2^Department of Nephrology, National Children's Medical Center, Children's Hospital of Fudan University, Shanghai, China

**Keywords:** hepatitis B, safety, vaccine, myocarditis, COVID-19

## Abstract

**Objectives:**

The hepatitis B vaccine comprises hepatitis B surface antigen (HBsAg) produced by transgenic yeast cells. There are few serious adverse events (SAE) reports after Hepatitis B vaccination.

**Methods:**

The authors searched the Chinese legal documents database for all SAE with Hepatitis B vaccination from January 2010 to January 2022.

**Results:**

All seven patients received yeast-derived recombinant hepatitis B vaccine. Three cases of myocarditis (death), 2 cases of interstitial pneumonia (death), and 2 cases of encephalitis. The mean time of onset of SAE was 8.3 ± 4.3 h after vaccination.

**Conclusion:**

The mechanism of vaccine-induced myocarditis may come from immune protein reactions. Based on the experience of Hepatitis B vaccine adverse events, we present new insights into the mechanism of myocarditis caused by the COVID-19 vaccine.

## Introduction

More than 800,000 people die from the Hepatitis B virus each year, and vaccination is the most cost-effective way to prevent the spread of the virus (1, 2). About 10% of the Chinese population carries the hepatitis B virus, and the Chinese government has implemented universal free hepatitis B vaccination since 2002 (3). Five enterprises in China produce hepatitis B vaccines, all recombinant hepatitis B vaccines. Hepatitis B vaccine is a milky suspension composed of purified virus surface antigen (HBsAg) and an aluminum adjuvant. And these vaccine preparations include sodium hydroxide, aluminum chloride, Disodium phosphate, sodium dihydrogen phosphate, and sodium chloride. Since 1999, thiomersal has been removed as a preservative from the Hepatitis B vaccine used in infant vaccinations (4). HBsAg is a hepatitis B virus capsid protein produced by recombinant yeast or CHO cells. HBsAg does not contain genetic material and pathogenicity. Recombinant hepatitis B vaccine can stimulate the body to produce protective antibodies and can be used to prevent all hepatitis B virus subtypes. The Hepatitis B vaccine showed well-established safety and effectiveness (5, 6). Clinical studies have shown that the most common adverse events are local pain, fever, and rash. These symptoms will subside within 3 days (7).

The whole course of Hepatitis B vaccination requires three doses given within a few days of birth, 1 month later, and 6 months later. Some rare serious adverse events (SAE) are associated with Hepatitis B vaccination. Besides, China still lacks a separate database for adverse events and data collection on serious adverse events related to vaccines. And serious adverse events are rarely reported for complex reasons (8). Therefore, it is a feasible supplementary method to obtain the vaccine adverse reaction from the public legal dispute database. We analyzed the available legal cases related to the hepatitis B vaccine and SAE from an open legal document database. The data from the real world may expand current public knowledge and improve patient safety with the hepatitis B vaccine.

## Methods

The data in this study came from the website database of China's court, https://wenshu.court.gov.cn. The authors searched the database from January 2010 to January 2022. Legal disputes include death after vaccination, prolonged hospitalization, and permanent disability. A panel of five experts evaluated the cause-and-effect relationship between vaccines and adverse reactions. The experts were senior specialists who have worked in clinical medicine, epidemiology, clinical laboratory, pharmacy, and forensic medicine for at least 3 years. The panel members evaluated causality by considering seven factors: time sequence, medicine information, dose-effect relationship, response pattern to drugs, reactivation, etiology, and combination of drugs. Then, this method evaluated outcomes as highly possible, possible, indeterminate, or impossible.

## Results

Seven severe adverse events related to the hepatitis B vaccine were retrieved from the database, five males and two females, with a ratio of 2.5:1. Six infants, were under 1 year of age, and one was an adult of 19 years. All were healthy before vaccination and had no hepatitis B or history of hepatitis B infection in their family history. Of all adverse events, 3 were myocarditis, 2 were meningitis, and 2 were interstitial pneumonia (Table 1).

**Table 1:** Summary of severe adverse reactions due to hepatitis B vaccine (*n* = 7).

**Gender**	**Age (years)**	**Vaccination times**	**Patient presentation/Diagnostic procedure**	**Diagnose**	**Prognosis**	**Causality**
Male	0.5	3	He developed a fever on the day of vaccination and was hospitalized for 2 days before being diagnosed with myocarditis and hemolytic uremic syndrome. He died 5 days later. The autopsy showed that the child died of myocarditis.	Myocarditis	Death	Highly possible
Female	0.6	3	She developed a fever and Rash 3 h after vaccination. The diagnosis was myocarditis. The patient's family refused to be hospitalized. Two days later, she died at home. The autopsy showed that the child died of myocarditis.	Myocarditis	Death	Highly possible
Male	19	4	He finished his vaccination in the morning and there was nothing abnormal after the vaccination. At night, he collapsed and died. The blood from his heart at autopsy was found: Myoglobin >3,000 μg/L, Creatine kinase isoenzyme > 300 μg/ L, Troponin> 10 μg/L.	Myocarditis	Death	Highly possible
Male	0.3	2	Two hours after he finished his vaccination, the parents found the child dead.	Interstitial pneumonia	Death	Highly possible
Female	0.5	3	She died in her sleep 11 h after being vaccinated.	Interstitial pneumonia	Death	Highly possible
Male	0.6	3	He developed fever, seizures, and other symptoms 8 h after the inoculation. After 3 months of hospitalization, the diagnosis was focal cerebral atrophy, encephalomalacia, gliosis, and cerebral palsy.	Encephalitis	Disability	Indeterminate
Male	0.6	3	He developed fever and febrile convulsion 9 h after vaccination. After 4 months of treatment, the diagnosis was: 1. Epilepsy: 2. Encephalitis: 3. Cerebral palsy.	Encephalitis	Disability	Indeterminate

Four infants and one adult died after the second vaccination. There were 3 cases of myocarditis and 2 cases of interstitial pneumonia. Their median time of death was 9 h after vaccination, ranging from 2 to 13 h. The onset of the adverse events was rapid, and there was no course of treatment in all the cases. The deceased patients had previously been in good health and had no heart disease or pneumonia history. The meeting of a panel of five senior medical experts (requested by the court) concluded that three cases of myocarditis were directly related to hepatitis B vaccination. Two patients could not determine the cause and effect of vaccine adverse reactions, which may be due to the long treatment time and slow progress of the disease.

Case 1: Less than 1 year-old. After his third dose of the hepatitis B vaccine, he had a fever for 3 days and hematuria for 2 days. He was diagnosed with myocarditis complicated by the hemolytic uremic syndrome. He died of multiple organ dysfunction syndromes 5 days later.

Case 2: Less than 1 year-old. She developed a fever and rash 3 h after her third vaccination. She was admitted to the hospital for myocarditis, but the parents refused the patient to be hospitalized. Two days later, she became sicker at home and died in the emergency room.

Case 3: Twelve hours after his fourth hepatitis B vaccination, he collapsed and died at home. At autopsy, the cardiac blood tests were myoglobin >3,000 μg/L, creatine kinase isoenzyme >300 μg/L, and troponin >10 μg/L.

Case 4: Less than 1 year-old. On the way home, he died 2 h after his second hepatitis B vaccination. The death was diagnosed as a result of acute respiratory failure caused by bilateral lung bronchopneumonia.

Case 5: Less than 1 year-old. She died 11 h after returning home after her third hepatitis B vaccination. The death was diagnosed as respiratory and circulatory failure due to interstitial pneumonia.

Case 6: Less than 1 year-old. He developed fever, cough, vomiting, diarrhea, and convulsions 8 h after the third dose. He was hospitalized for 3 months with focal brain atrophy, encephalomalacia, and gliosis. His final diagnosis was encephalitis sequelae and disability.

Case 7: Less than 1 year-old. He developed fever 9 h after the third dose. Oral ibuprofen was not practical, and he had one convulsion. After 4 months in hospital, he was diagnosed with epilepsy, and cerebral palsy, which resulted in a disability.

## Discussion

The smallpox vaccine is recognized to induce myocarditis with an incidence of 16 cases per 100,000 (9). Our study reported three cases of myocarditis induced by hepatitis B vaccination to raise the attention of this potential severe adverse event after vaccination. According to the China Health Statistics Yearbook, 185 million babies were vaccinated against the Hepatitis B vaccine. Therefore, seven severe cases represented the incidence of serious adverse events was 4 per 1 million. We may underestimate the incidence rate, but it is consistent with the phenomenon that only some adverse events cause legal controversy.

Why does myocarditis cause rapid infant death in a short period? We think there are three possible reasons. First, fulminant myocarditis is a hazardous disease, with a pediatric patient's mortality rate of more than 48% (10). The Pathophysiology of heart failure in children is markedly different from that in adults (11). Second, the patient is too late to be rescued in time. The cardiovascular adverse effects of the vaccine do not occur immediately after the injection. Third, early adverse events (crying, shortness of breath) in infants who received subsequent vaccinations were ignored based on the excellent safety of the first dose. In addition, our study showed a higher incidence of myocarditis in men than in women (2:1). This appearance may be related to the physiological characteristics of a male. Men also have a higher incidence of other myocarditis, with more severe heart symptoms (12).

Hepatitis B vaccine myocarditis is associated with allergic vasculitis and may subside with corticosteroid therapy (13). In all of the cases in our study, adverse events occurred immediately after the second dose of the vaccine. It is suggested that hepatitis B vaccine-induced myocarditis may be a drug-induced allergic syndrome. Accordingly, severe autoimmune adverse events following Hepatitis B vaccine vaccination have been reported in the literature (14). There is a causal relationship between the hepatitis B vaccine and autoimmune diseases, including rheumatoid arthritis, myelitis, optic neuritis, and nephritis (15). And the Hepatitis B vaccine is positively associated with an increased incidence of child arthritis (16). Two cases in this study with childhood encephalitis caused by the hepatitis B vaccine may also be related to autoimmune disease.

The mRNA-Based COVID-19 vaccine is similar to the hepatitis B vaccine in the following perspectives. First, both vaccine's substrates induce human immune responses and are partly identical in structure (HBsAg protein *vs*. SARS-CoV-2 S protein). For example, hepatitis B proteins presented antigenic properties that can cause a putative protective effect against COVID-19 (17). Second, all the adverse occurs after the second dose, regardless of the vaccine source (18, 19). Recently, many studies have shown that multiple mRNA-Based COVID-19 vaccines can cause myocarditis (20, 21). On the myocarditis reports of 1,626 cases, 82% occurred after the second vaccination dose (20). Another study showed that all instances of juvenile myocarditis occurred 4 days after the second injection (22). Third, the cases in this study were all sudden myocarditis deaths, similar to those caused by SARS-CoV-2. Therefore, we hypothesized that the first vaccine established immune system memory, and then the second vaccine caused acute allergies, including myocarditis or other autoimmune diseases. Interestingly, a new COVID-19 Vaccine, Novavax, is clearly stated in its specifications: Clinical trials data provide evidence for increased risks of myocarditis (23).

Rare heart death cases were reported in patients after their mRNA COVID-19 vaccination (21). We suspected that the mild symptoms of the mRNA vaccine might be related to the slow release of SARS-CoV-2 S protein with mRNA vaccines. The Hepatitis B vaccine contains protein injected directly into the body and can induce acute allergies. In contrast, mRNA-Based COVID-19 vaccine takes a while to translate the mRNA into virus protein, which acts as a “slow-release” process, resulting in milder symptoms and few sudden deaths. The limitation of this study is that our legal documents only report a small number of cases and may have missed other patients.

## Conclusion

In this report, we summarize each patient's clinical course and evaluation. Despite these rare adverse events, the benefit of universal vaccination against hepatitis B still outweighs the risk at this point. Hepatitis B vaccine-induced serious adverse events are rare, and myocarditis may be related to an autoimmune reaction. The vaccination after the first dose is associated with a higher risk of SAE than the first dose. The similarity between the hepatitis B vaccine protein and the COVID-19 vaccine-derived protein, we should pay close attention to the vaccine autoimmune response, especially myocarditis.

## Data availability statement

The raw data supporting the conclusions of this article will be made available by the authors, without undue reservation.

## Ethics statement

The studies involving human participants were approved by Medical Ethics Committee of Children's hospital of Fudan University in Shanghai, China (No. 2020-187). Written informed consent from the participants' legal guardian/next of kin was not required to participate in this study in accordance with the national legislation and the institutional requirements.

## Author contributions

JL and XZ requested the data sets and drafted the manuscript. HX and ZL were involved in the study conception and design and critically reviewed the draft manuscript. All authors approved the final manuscript as submitted and agreed to be accountable for all aspects of the work.

## Funding

This work was supported by National Natural Science Foundation (grant number 81874325) and the Scientific Research Project of Science and Technology Commission of Shanghai Municipality (grant numbers 19DZ1910604, 19XD1400900, and 18DZ1910604).

## Conflict of interest

The authors declare that the research was conducted in the absence of any commercial or financial relationships that could be construed as a potential conflict of interest.

## Publisher's note

All claims expressed in this article are solely those of the authors and do not necessarily represent those of their affiliated organizations, or those of the publisher, the editors and the reviewers. Any product that may be evaluated in this article, or claim that may be made by its manufacturer, is not guaranteed or endorsed by the publisher.
